# Polymyxins–Curcumin Combination Antimicrobial Therapy: Safety Implications and Efficacy for Infection Treatment

**DOI:** 10.3390/antiox9060506

**Published:** 2020-06-09

**Authors:** Chongshan Dai, Yang Wang, Gaurav Sharma, Jianzhong Shen, Tony Velkov, Xilong Xiao

**Affiliations:** 1 Department of Veterinary Pharmacology and Toxicology, College of Veterinary Medicine, China Agricultural University, No.2 Yuanmingyuan West Road, Beijing 100193, China; wangyang@cau.edu.cn (Y.W.); sjz@cau.edu.cn (J.S.); 2 Advanced Imaging Research Center, University of Texas Southwestern Medical Center, Dallas, TX 75390, USA; Gaurav.Sharma@utsouthwestern.edu; 3 Department of Pharmacology & Therapeutics, Faculty of Medicine, School of Biomedical Sciences, Dentistry and Health Sciences, the University of Melbourne, Parkville 3052, Australia; Tony.Velkov@unimelb.edu.au

**Keywords:** polymyxin B, colistin, curcumin, oxidative stress, mitochondrial dysfunction

## Abstract

The emergence of antimicrobial resistance in Gram-negative bacteria poses a huge health challenge. The therapeutic use of polymyxins (i.e., colistin and polymyxin B) is commonplace due to high efficacy and limiting treatment options for multidrug-resistant Gram-negative bacterial infections. Nephrotoxicity and neurotoxicity are the major dose-limiting factors that limit the therapeutic window of polymyxins; nephrotoxicity is a complication in up to ~60% of patients. The emergence of polymyxin-resistant strains or polymyxin heteroresistance is also a limiting factor. These caveats have catalyzed the search for polymyxin combinations that synergistically kill polymyxin-susceptible and resistant organisms and/or minimize the unwanted side effects. Curcumin—an FDA-approved natural product—exerts many pharmacological activities. Recent studies showed that polymyxins–curcumin combinations showed a synergistically inhibitory effect on the growth of bacteria (e.g., Gram-positive and Gram-negative bacteria) in vitro. Moreover, curcumin co-administration ameliorated colistin-induced nephrotoxicity and neurotoxicity by inhibiting oxidative stress, mitochondrial dysfunction, inflammation and apoptosis. In this review, we summarize the current knowledge-base of polymyxins–curcumin combination therapy and discuss the underlying mechanisms. For the clinical translation of this combination to become a reality, further research is required to develop novel polymyxins–curcumin formulations with optimized pharmacokinetics and dosage regimens.

## 1. Introduction

**Figure 1:**
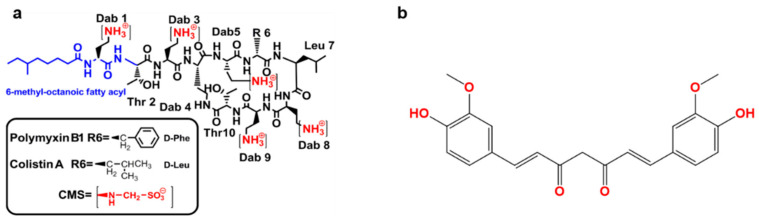
The structure of polymyxin B and colistin (**a**) and curcumin (**b**). CMS—colistin methanesulfonate; Thr—threonine; Leu—leucine; Phe—phenylalanine; Dab—α,γ- diaminobutyric acid.

The World Health Organization [1] has highlighted antimicrobial resistance as a major global health concern [1]. As no new classes of antibiotics will be available for Gram-negative ‘superbugs’ in the near future, we have to develop novel polymyxin combination therapies [2]. Polymyxins were firstly discovered in 1947 from different species of *Bacillus polymyxa* [3]. There are five members in the polymyxins family, i.e., polymyxin A, B, C, D and E (also named colistin). Out of these, only polymyxins B and colistin are used in clinical practice, as agents against multi-drug resistant (MDR) Gram-negative pathogens, in particular *Pseudomonas aeruginosa* (*P. aeruginosa*) *Acinetobacter baumannii* (*A. baumannii*) and *Klebsiella pneumoniae* (*K. pneumoniae*). They differs by only one amino acid (Figure 1a) [4,5,6,7,8,9,10,11].

Unlike polymyxin B, which is available in the clinic as the sulfate salt, colistin is used in the clinic as an inactive pro-drug, colistin methanesulfonate (CMS) (Figure 1a) [12,13]. Polymyxin B is available for intravenous, oral and topical use and CMS for parenteral use; both can be delivered by inhalation [14]. Plasmid carried mobilized colistin resistance (mcr) gene-mediated polymyxin resistance has been increasingly reported in MDR Gram-negatives worldwide. Polymyxin resistance implies a total lack of antibiotics against Gram-negative ‘superbugs’, without the availability of novel antibiotics in the near future, the development of superior polymyxin combinations to combat a ‘post-antibiotic era’ is paramount [15,16]. This has brought the effectiveness of polymyxin monotherapy into question. Unfortunately, only increasing the dose of polymyxins to overcome resistance is not an option due to toxic side effects (i.e., nephrotoxicity and neurotoxicity) [17]. Previous clinical observations showed that the rates of nephrotoxicity occurred in approximately 60% after patients received colistin or polymyxin B therapy [18]. Based on the narrow therapeutic indices of polymyxins, a key strategy for overcoming resistance and concomitantly ablating unwanted side effects is combining polymyxins with other agents. Although polymyxin-antibiotic combinations exhibit well-confirmed synergistic effects against bacterial growth in vitro [19,20]. The clinical findings from randomized, controlled prospective trials have shown that these combinations have no increased benefits compared to their respective monotherapies [19,20]. Moreover, some polymyxin-antibiotic combinations may increase the risk of renal failure (e.g., colistin–vancomycin combination) [20].

Secondary metabolite natural products have been at the forefront of drug candidates for the treatment of cancer, infection and neurodegenerative diseases for decades [21]. Natural products have been the source of most of our antibacterial armamentarium and efforts over the last 30 years [22]. Recent studies have shown that combining polymyxins with curcumin has many pharmacological and toxicological benefits over monotherapy with each compound per se including: (1), the in vitro synergy against various strains of MDR bacteria [23], including polymyxin resistant isolates [24]; (2), protection against polymyxin-induced nephron- and neuro-toxicity [25,26,27]; (3), curcumin supplementation improves recovery from the bacterial infection or LPS—induced inflammatory response or sepsis [28,29,30,31,32,33]. Importantly, human clinical trials indicated that curcumin displays good safety and tolerability [34,35,36]; oral administration of curcumin at eight grams per day over three months or a single oral dose of 12 grams has no marked adverse effect [37,38].

In combination with curcumin, polymyxins hold great promise as combination therapy for infections caused by MDR Gram-negative pathogens. In this review, we summarize the current understanding of the underlying molecular mechanisms on nephro- and neuroprotection and synergistic effects on bacterial growth of this novel combination.

## 2. Curcumin

Curcumin, also known as diferuloylmethane, E100 or natural yellow 3 (International Union of Pure and Applied Chemistry (IUPAC) name is (1E, 6E)-1,7-bis(4-hydroxy-3-methoxyphenyl)-1,6-heptadiene-3,5-dione, Figure 1b), is a natural polyphenol extracted from the rhizome of *Curcuma longa* [39]. Curcumin displays many beneficial pharmacological actives, such as anti-inflammatory, anti-oxidant, antitumor and notably, antimicrobial activities [24,40,41,42,43]. The direct antibacterial activities of curcumin have been widely studied [44,45]. It is purported that the anti-inflammation and antioxidant abilities may contribute to the indirect antibacterial activities of curcumin by modulating the interaction of host cells with bacteria or via increasing the potential loading dose of a combination antibiotic drug by inhibiting the unwanted adverse effects.

### 2.1. Antibacterial Activity of Curcumin

Curcumin exhibits antibacterial activities against both Gram-negative and Gram-positive bacteria, including MDR and polymyxin-resistant isolates [44,45]. Curcumin has been shown to disrupt filamenting temperature-sensitive mutant Z (FtsZ) protofilament activity that orchestrates bacterial cell division [46]. In contrast to its action on mammalian cells, in bacteria, curcumin induces the production of reduced reactive oxygen species (ROS), including superoxide anions (O2^•−^), hydroxyl radicals (^•^OH) and hydrogen peroxide (H_2_O_2_), which kills bacteria by damaging proteins, lipids and DNA [47,48,49]. ROS-mediated phototoxicity also contributes to the antibacterial activities of curcumin [50]. Curcumin inhibits the expression of biofilm initiation genes and quorum sensing (QS) genes, and downregulates the virulence factors including the production of acyl-homoserine lactone (HSL), pyocyanin biosynthesis and elastase/protease activity [51,52]. A study from Mun et al., showed that the minimum inhibitory concentration (MIC) values of curcumin against 10 strains of *Staphylococcus aureus* (*S. aureus*) ranged from 125 to 250 μg/mL [53]. Moreover, curcumin displays significant antibacterial activity against the stomach ulcer-causing pathogen *Helicobacter pylori* (*H. pylori*) with MIC values ranging from 5 to 50 μg/mL against 65 clinical isolates [54]. Another study with a crude aqueous rhizome extract of *Curcuma longa* showed MIC value of 4 to 16 μg/mL against strains of *S. epidermis* ATCC 12228, *S. aureus* ATCC 25923, *K. pneumonia* ATCC 10031 and *E. coli* ATCC 25922 [55]. The rhizome extract of *Curcuma longa* includes primarily curcumin and other derivative compounds such as curdione, isocurcumenol, curcumenol, curzerene, β-elemene, germacrone and curcumol [56]. Notwithstanding its direct antibacterial activities, curcumin displays potent synergistic effects when combined with antibiotics (e.g., oxacillin, ampicillin, polymyxin B and norfloxacin) [23,53].

The therapeutic potential of curcumin is limited owing to its poor oral bioavailability and insufficient solubility in aqueous solvents. Therefore, oral curcumin often present poor absorption, fast metabolism and quick systemic elimination in animal experiment and human clinical trial [44,45,52]. Researchers have attempted to solve these problems by developing new drug delivery methods such as liposomes, solid dispersion, microemulsion, micelles, nanogels and dendrimers [52]. For example, the poly (lactic-co-glycolic acid) (PLGA) polymeric nanocapsules for the delivery of curcumin can enhance its solubility (increase by ~1500-fold, compared to free curcumin) and antibacterial activity (MIC values decrease by ~2-fold, compared to free curcumin) [57]. In another example, curcumin–β-cyclodextrin nanoparticle complex formation exhibited a potent bactericidal activity by increasing the production of ROS and inhibiting electron transport; polyelectrolyte-coated monolithic nanoparticle formation exhibited a potent bacteriostatic effect by increasing membrane depolarization and reducing ATP concentrations [58].

### 2.2. Effect of Curcumin on Bacteria or Its Toxin-Induced Inflammatory Response

The curcumin structure has functional groups that contribute to its ability to scavenge ROS including phenyl rings, carbon–carbon double bonds and β-diketone structures [59]. Curcumin also directly targets a plethora of pathways that play important roles in the inflammatory response, oxidative stress and cell death, including cyclooxygenase 2 (COX-2), lipoxygenase, protein kinase B (PKB, also named Akt), toll-like receptor (TLR)-4, nuclear factor erythroid 2-related factor 2 (Nrf2), glycogen synthase kinase (GSK)-3β, phosphorylase-3 kinase, focal adhesion kinase, glutathione, xanthine oxidase, pp60 src tyrosine kinase and ubiquitin isopeptidase [60,61,62,63]. These signals and/or pathways also play critical roles in response to bacterial infection or its pathogenic factors (e.g., α-hemolysin and lipopolysaccharide [LPS])-mediated pathology [29,64,65,66]. For example, curcumin has an inhibitory effect on NF-*κ*B activation, the release of interleukin (IL)-8 and matrix metalloproteinase-3 and metalloproteinase-9 activity induced by *H. pylori* infection in the stomach of mice or cell culture with a dose-dependent manner [67,68]. Curcumin can also inhibit LPS-induced activation of NOD-, LRR- and pyrin domain-containing protein 3 (NLRP3) inflammasome and the secretion of IL-1β and high mobility group box 1 (HMGB1) [69,70,71].

## 3. Synergistic Antibacterial Effects of the Polymyxin in Combination with Curcumin

The putative primary mechanism of action of polymyxins is the disruption of the Gram-negative outer membrane through an initial electrostatic interaction that results in cation displacement (Ca^2+^ and Mg^2+^) and subsequent binding to the lipid A component of LPS, leading to leakage of the cytoplasmic content and ultimately causing cell death [72]. As a secondary killing effect, polymyxins induce ROS [73]. The inhibition of bacterial respiration has also been associated with the bacterial killing action of polymyxins [74].

Polymyxin resistance is conferred by mcr-mediated structural modifications of LPS that act to reduce the negative charge of the outer membrane, which in turn repels the polymyxin molecule [75]. The two main LPS modifications conferring polymyxin resistance are the addition of phosphoethanolamine (PetN) and 4-amino-4-deoxy-L-arabinose (AraN) to the lipid A [76]. Recently, plasmid-mediated colistin resistance was reported in *Enterobacteriaceae* due to the PetN transferase mcr-1 [15].

**Figure 2:**
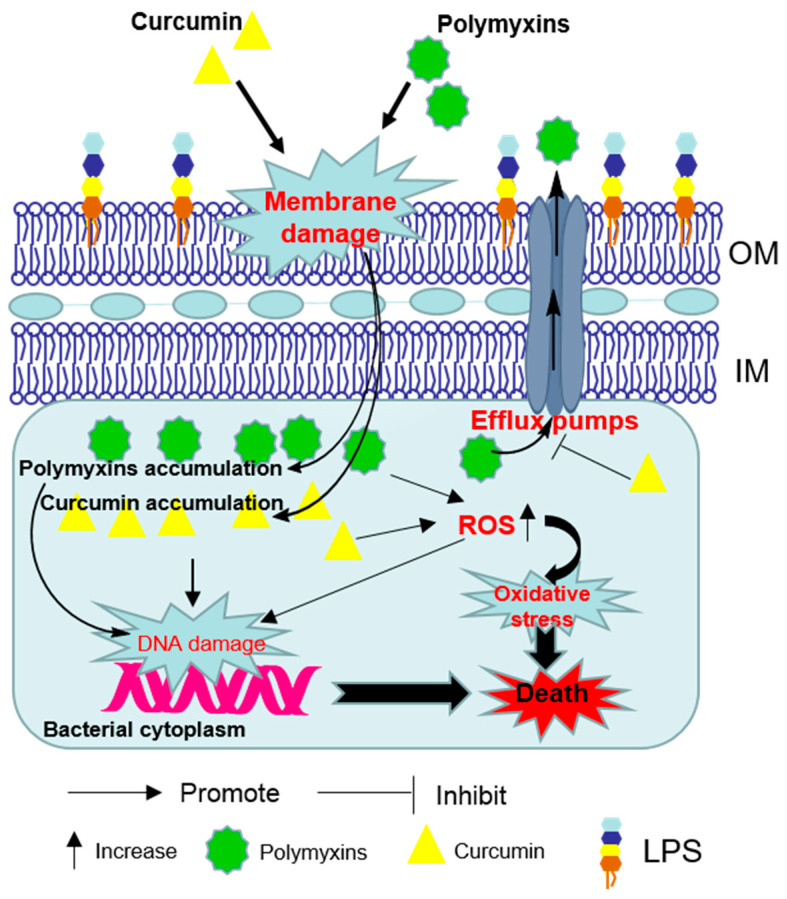
Schematic diagram depicting the synergistic bacterial killing mechanism of the polymyxins–curcumin combination. Polymyxins permeabilize the Gram-negative outer membrane and thereby promote curcumin enter the bacterial cell. Bacterial cell death finally ensues via reactive oxygen species (ROS) production, oxidative stress and DNA damage. Curcumin may also overcome the polymyxin-resistance by disturbing the activities of efflux pumps. OM—outer membrane; IM—inner membranes; LPS—lipopolysaccharide.

In vitro time-kill curves for polymyxin B in combination with curcumin showed a marked synergetic effect against antibiotic-susceptible and—resistant Gram-positive (*Enterococci*, *S. aureus* and streptococci) and Gram-negative (*A. baumannii*, *E. coli*, *P. aeruginosa* and *S. maltophilia*) bacterial isolates associated isolated from traumatic wound infections [23]. The synergistic effect may result from the ability of polymyxin to permeabilize the outer membrane which facilitates the access of greater concentrations of curcumin to its intracellular targets (Figure 2) [24]. Curcumin may also overcome the polymyxin-resistance by inhibiting the activities of efflux pumps where located in outer membrane (OM) or inner membranes (IM) [24].

## 4. Polymyxin-Induced Nephrotoxicity and Protective Effect of Curcumin

Nephrotoxicity is the major dose-limiting factor of polymyxins in clinical practice and it occurs in up to 60% of patients after intravenous administration [18]. Clinical manifestations include a decrease in creatinine (CRE) clearance, as well as proteinuria, oliguria (low output of urine) and cylindruria (presence of casts in the urine) [18,77,78]. Pathologic characteristics of colistin or polymyxin B-induced nephrotoxicity were included tubular dilation and tubular epithelial cell vacuolation and necrosis, tubular casts and inflammatory cell infiltration [79,80,81]. In clinical practice, serum CRE and blood urea nitrogen (BUN) are usually employed as the biomarkers of renal function. However, their use as the biomarkers has some limitations in polymyxin therapy, such as dependence on nutrition, age, sex and body mass and is likely to reflect already advanced damage. Studies in animal models showed that kidney injury molecule 1 (KIM-1), Cystatin C and α-glutathione *S*-transferase may be more reliable markers than CRE or BUN to monitor renal function during polymyxin therapy [82,83].

**Figure 3:**
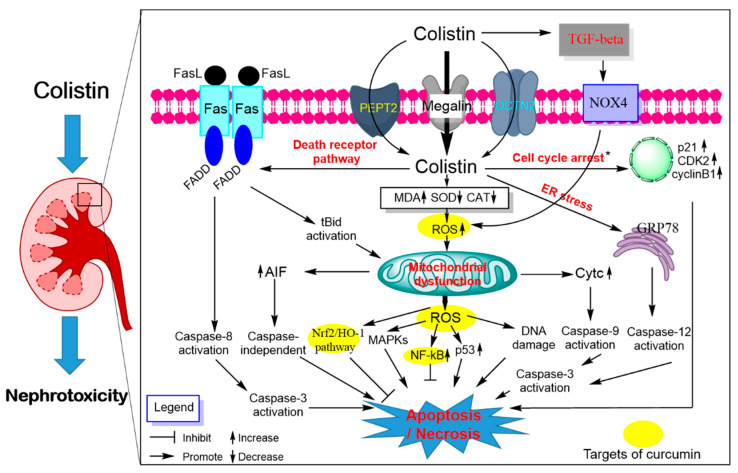
Pathways involved in polymyxin-induced nephrotoxicity and the potential nephro-protective mechanism of curcumin. Colistin extensively accumulates in kidney tubular cells via receptor-mediated uptake, megalin, peptide transporter 2 (PEPT2) and the carnitine/organic cation transporter 2 (OCTN2). Intracellular colistin accumulation in kidney tubular cells induces the production of ROS, mitochondrial dysfunction and triggers a series of signaling cascades (e.g., cell cycle arrest, p53, nuclear factor kappa B [NF-κB] and mitogen-activated protein kinase [MAPK], nuclear factor erythroid 2-related factor 2 [Nrf2]/heme oxygenase-1 [HO-1] pathways). The activation of the transforming growth factor (TGF)-β/nicotinamide adenine dinucleotide 3-phosphate oxidase-4 (NOX-4) pathway contributes to oxidative stress by promoting ROS production. All three major apoptosis pathways (e.g., mitochondrial, death receptor and endoplasmic reticulum pathways) participated in colistin-induced nephrotoxicity. Curcumin treatment improved colistin-induced nephrotoxicity by targeting the NF-kB mediated inflammatory response, oxidative stress and upregulating the antioxidant Nrf2/HO-1 pathways.

Our understanding of the molecular mechanisms underlying polymyxin-induced nephrotoxicity have significantly advanced in the past 15 years owing to the pioneering work of the Li group from Monash University, Australia [84,85,86,87,88,89,90,91,92,93,94,95,96]. Their recent studies have revealed significant renal accumulation of polymyxins using immunostaining, fluorescence microscopy, mass spectrometry imaging and X-ray fluorescence microscopy (XFM) [84,85,86,87]. Predominant accumulation of polymyxin B was distinct in the renal cortex, in particular the renal proximal tubular cells, but much less in the distal tubular cells [84,85]. Moreover, polymyxins substantially accumulate in proximal tubular cells via receptor-mediated endocytosis mainly by megalin [97,98], human peptide transporter 2 (PEPT2, *syn.* SLC15A2) [91,96] and the carnitine/organic cation transporter 2 (OCTN2, *syn.* SLC22A5) [99] (Figure 3). Inhibitors of these receptors were shown to block the uptake of polymyxins and then attenuate colistin or polymyxin B- induced nephrotoxicity in mice [91,97,100].

Polymyxin treatment can lead to DNA damage and apoptotic cell death in renal tubular cells in the cultured cell model or in vivo animal model [93,101,102,103] (Figure 3). Our group has been pioneering this area in reporting a series of studies showing that polymyxin B or colistin-induced apoptosis involved the death receptor (upregulation of Fas, FasL and Fas-associated death domain [FADD]), mitochondrial (downregulation of B-cell lymphoma 2 [Bcl-2] and upregulation of cytochrome C [CytC] and bcl-2-like protein 4 [Bax] and endoplasmic reticulum (upregulation of activating transcription factor 6 [ATF6], glucose-regulated protein 78 [GRP78], caspase-12 and growth arrest and DNA damage-inducible gene 153 [GADD153/CHOP]) pathways in cultured renal tubular cells [101] and the kidney tissue of mouse [93]. Polymyxin B or colistin treatment can induce loss of mitochondrial membrane potential, morphology changes and the generation of ROS mediated oxidative damage in a concentration-dependent manner [93,101]. Significant Elevated protein expression levels of the p53, cyclin-dependent kinase 2 (CDK2) and phosphorylated Jun N-terminal kinase (JNK) and p38 mitogen-activated protein kinase (MAPK) and autophagy were detected in kidney tissues of the colistin-treated mice [93]. The lipid peroxidation marker malondialdehyde (MDA), nitrative stress-related nitric oxide (NO) and inducible nitric oxide synthase activities and inflammation response were significantly increased in the kidney tissues from colistin-treated mice [80,104]. Additionally, the activation of transforming growth factor (TGF)-β/nicotinamide adenine dinucleotide 3-phosphate oxidase-4 (NOX-4) pathway contributes to the production of ROS in kidneys of mice; inhibition of TGF-β expression markedly attenuated colistin-induced renal damage in mice, indicating that TGF-β/NOX-4 pathway may play a critical role in colistin-induced nephrotoxicity [105].

Curcumin has been shown to protect against colistin-induced nephrotoxicity via inhibition of oxidative stress, inflammation and apoptosis. A previous study reported that curcumin supplementation (e.g., at 200 mg/kg/day for 3 days) markedly protects against glycerol-induced acute kidney injury by inhibiting oxidative damage and activating Nrf2 pathway in rats [106]. A similar molecular mechanism has also been detected in the protection of curcumin against gentamicin-induced nephrotoxicity in rats [107]. Consistent with these findings, curcumin supplementation (e.g., at 200 mg/kg/day for 6 days) can also markedly improve colistin-induced nephrotoxicity via the inhibition of oxidative stress, apoptosis, NO signaling and inflammatory response (i.e., decreases the tumor necrosis factor-α [TNF-α], interleukin-6 [IL-6] in the kidney tissue) in a rat model [27]. Our recent data reveal that curcumin after oral administration at 50 and 200 mg/kg/day for 7 days can be detected and accumulated in multiple organs of mice, including kidney tissues [25]. It was shown that curcumin can promote Nrf2 translocation into nuclei through modification of cysteine sulfhydryl groups in Kelch-like ECH-associated protein 1 (Keap1), a principal negative regulator of Nrf2 in the cytoplasm. The binding of Nrf2 and antioxidant responsive element (ARE) in nuclei activated the transcription activity of ARE that mediates the expression of antioxidant gene such as heme oxygenase-1 (HO-1), CAT and SOD [108]. Indeed, curcumin can directly activate Nrf2/ARE pathway and protects against oxidative stress-mediated apoptotic cell death [109]. Therefore, the activation of Nrf2/HO-1 may contribute to the protective effect of curcumin against colistin–oxidativedamage in the kidney tissues. Besides, it has been reported that curcumin supplementation can protect some drugs (e.g., cisplatin, glycerol and doxorubicin) or toxins (e.g., potassium dichromate, maleate and arsenic)-induced nephrotoxicity by inhibiting oxidative stress, ER stress, inflammatory response, NO pathway, ferroptosis, p53 pathway, MAPK, AMP-activated protein kinase (AMPK) pathways, Akt pathway or activation of autophagy [106,110,111,112,113,114,115,116]. The previous study showed that the antioxidant ascorbic acid can protect against colistin-induced nephrotoxicity and cellular apoptosis. Meanwhile, ascorbic acid altered the pharmacokinetics of colistin in a rat model, the total body clearance of colistin decreased from 3.78 ± 0.36 mL/min/kg (colistin alone group) to 2.46 ± 0.57 mL/min/kg (ascorbic acid + colistin co-treatment group), and the half-life of plasma colistin concentration increased from 1.20 ± 0.23 (colistin alone group) h to 3.91 ± 0.42 h (ascorbic acid + colistin co-treatment group) [94].

## 5. Colistin-Induced Neurotoxicity and Protective Effects of Curcumin

Neurotoxicity caused by colistin has been observed in a dose-dependent manner that may be associated with colistin accumulation in the nerve tissues [25]. In contrast to nephrotoxicity, the incidence of neurotoxicity associated with the use of polymyxins is less commonly reported in the literature, despite being a significant clinical complication [117]. Patients who received intravenous polymyxins have been reported to present with potential neurological symptoms including dizziness, vertigo, visual disturbances, hallucinations, confusion, seizures, ataxia, facial and peripheral paresthesias [117,118,119]. Mild neurotoxicity symptoms (e.g., paresthesias) is more frequent than others, especially in elderly patients, but are often ignored due to the lack of effective assessment protocols [117,118,119,120]. An early study reported that paresthesias and ataxia as the major neurotoxicity symptoms occur about in the 29% of patients that received intravenous administration of CMS at the dose of over 5 mg/kg/day (range from 5.7 to 8.0 mg/kg/day) [121]. A recent study indicated that intravenous administration of high doses of polymyxin B (range from 3 to 6 mg/kg/day) may increase the clinical outcome, but the neurotoxicity events also increased in patients [122].

Investigation of polymyxin uptake into the central nervous system (CNS) and neuronal cells is a critical aspect of understanding the neurotoxicity. The blood–brain barrier (BBB) is the “first barrier” that mediates the uptake of the drug into neuronal cells in the brain tissues, and it only allows small molecules that both have the low molecular mass (<450 Da) and high lipid solubility to pass [123]. The tight junctions of the interendothelial domains limit the passage of large hydrophilic molecules cross the BBB and this is considered as the main reason underlying the low BBB penetration of polymyxins (molecular mass of colistin and polymyxin B are ~1155 Da and ~1, 203, respectively) [124]. This may also explain the higher incidence in peripheral nervous system (PNS)- than CNS- mediated polymyxin neurotoxicity in clinic [117,119,125]. It is negligible in the brain uptake of colistin when healthy mice were received a single intravenous dose (5 mg/kg) or subcutaneous dose (40 mg/kg) [124,126,127,128]. However, Wang et al., showed that the continuous administration of intravenous colistin sulfate at 15 mg/kg/day for 7 days to mice significantly increased the colistin concentration in brain tissues without BBB damage [129]. Consistently, our group showed that colistin could be accumulated in nerve tissues including the brain, cerebellum and sciatic nerve with the abnormal neurobehavioral and electrophysiology changes when mice were intraperitoneally injected with colistin sulfate at 18 mg/kg/day for 7 days [25,120,130,131,132]. It has also been demonstrated that the intravenous administration of LPS can increase the BBB transport of colistin in mice, indicating that systemic inflammation (e.g., CNS infections and sepsis) may increase the colistin concentrations in the brain tissues [124,126,127,128]. Our in vitro studies have shown that the absorption of colistin into mouse primary cortical neuronal cells is in a concentration-dependent manner [133]. As above mentioned, megalin, PEPT2, and OCTN2 can mediate the uptake of colistin into kidney cells [91,97,99,134]. Notably, the expressions of megalin, PEPT2, and OCTN2 are also detected in neuronal cells [135,136,137], and as such may mediate polymyxin uptake into neurons.

Due to its high oxygen use and high content of polyunsaturated fatty acids, the CNS was well known to be particularly vulnerable to oxidative damage [138]. Mitochondria play a critical role in maintaining basic cellular functions, including cellular energy metabolism, ATP production and triggering cellular apoptosis and autophagy [138]. We have shown that mitochondria in the cerebrum and sciatic nerve tissues showed abnormal ultrastructure (e.g., disruption of cristae and swelling) and functions when mice were intravenously injected with colistin at 15 mg/kg/day for 7 days [131,139]. Mitochondrial dysfunction was also detected in primary chick neuronal cells when they were treated with colistin at 4.15 and 8.3 μg/mL for 24 h [140]. Excessive ROS from mitochondria can lead to lipids, proteins and DNA damage and ultimately results in cell death [141]. Our recent study showed that colistin treatment (200 μM for 24 h) significantly induced the increase in the levels of intracellular ROS with a decrease in the levels of glutathione (GSH) levels and the activity of the antioxidant enzymes SOD and CAT in the N2a neuronal cells [142]. These data lend substantial evidence that oxidative stress and mitochondria dysfunction play critical roles in colistin-induced neurotoxicity. Moreover, Nrf2 is suggested as the “housekeeping” gene in response to oxidative stress. Nrf2 activation can transcriptionally activate the expression of several phase II enzymes, including HO-1, SOD and CAT [92]. Not surprisingly, the upregulation of Nrf2/HO-1 pathway in N2a cells and the cerebral cortices of mice treated with colistin were detected [131,132,133,142]. These evidences suggested that the activation of Nrf2/HO-1 plays a protective role in polymyxins-induced neurotoxicity.

**Figure 4:**
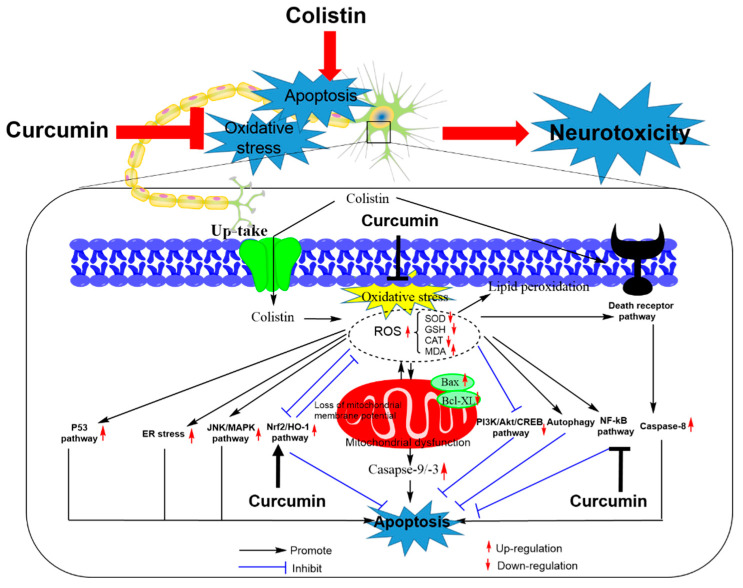
Schematic diagram depicting the major apoptosis pathways involved in colistin-induced neurotoxicity and the potential targets of curcumin. Colistin-induced apoptosis involves oxidative stress, the activation of death receptor and mitochondrial pathways by upregulating the Bax/Bcl2 ratio, inducing the loss of membrane potential and production of reactive oxygen species [ROS] and activating caspases-3, -8 and -9. Colistin-induced apoptosis also involved the activation of p53, nuclear factor kappa B (NF-κB), ER stress, c-Jun *N*-terminal kinases (JNKs)/MAPK pathways and the inhibition of the PI3 K/Akt/CREB pathway. The activation of autophagy and Nrf2/HO-1 pathways inhibits colistin-induced apoptosis. Curcumin ameliorates colistin-induced apoptosis and neurotoxicity by targeting the Nrf2/HO-1, NF-κB and oxidative stress pathways.

It has been reported that the main pathways of colistin-induced apoptosis in neuronal cells involve the intrinsic mitochondrial and extrinsic death receptor pathways [142,143,144,145]. Colistin treatment can cause an increase in the Bax/Bcl-2 ratio and cytochrome C (CytC) release, in cascade to triggering the activations of caspases-3 and -9 and inducing apoptotic death in N2a and PC12 neuronal cells [142,143,144]. Similar in kidney cells, colistin treatment of PC12 cells also exhibited the increased expression of Fas and Fas-L, followed to activate caspase-8 through the FADD [145]. Notably, the pan-caspase inhibitor Z-VAD-FMK can partially block colistin-induced apoptotic death in N2a neuronal cells, highlighting the role of caspases activation in intrinsic and extrinsic apoptotic pathways [142]. In a mouse model, the marked increase of GRP78, an ER stress marker, was detected in the cerebral cortical tissues, indicated the ER stress may also contribute to colistin-induced neurotoxicity [132]. Our recent studies showed that colistin treatment (200 μM) of N2a neuronal cells downregulated the expression of mammalian target of rapamycin (mTOR) and phospho (p)-p70S6 kinase (p70s6k) and upregulates unc-51 like autophagy activating kinase 1 (ULK1) expression, suggesting that mTOR inhibition-mediated autophagy is triggered in the process of colistin-induced apoptosis in neuronal cells [128]. In addition, colistin-induced neuronal cell death also involves p53, MAPK and PI3 K/Akt/c-AMP response element-binding (CREB) pathways, and their roles are shown in Figure 4 [26,27,78,81,132,142,146,147,148,149,150].

**Figure 5:**
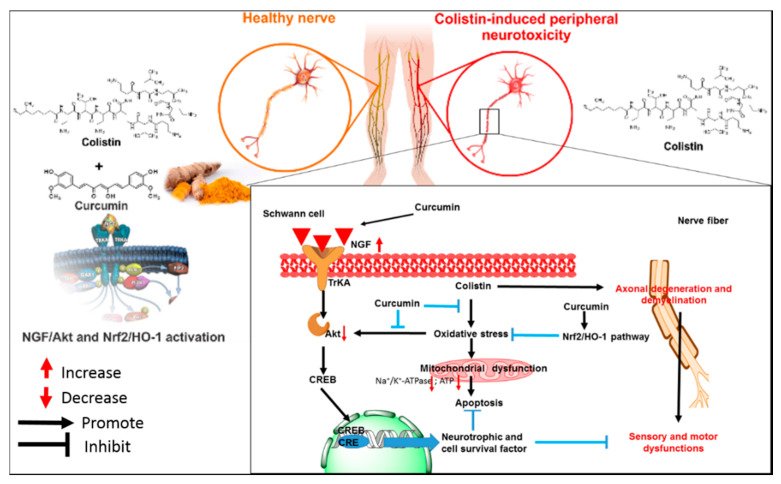
Schematic diagram depicting the putative action of curcumin against colistin-induced peripheral neurotoxicity. Oral administration of curcumin improves colistin-induced dysfunctional motor and sensory symptoms by inhibition of oxidative stress and activation of the NGF/Akt and Nrf2/HO-1 pathways in the sciatic nerve tissues of mice. The figure is a revision from Dai. et al., [26] and [154].

Curcumin supplementation effectively protects against colistin treatment-induced neurotoxicity in vitro (e.g., mouse primary and N2a neuronal cells) and animal models (e.g., mice and rats) (Figure 4) [25,26,27]. Curcumin indicated the potential application as a neuroprotective agent with an ability to cross the BBB [40,151,152]. Our in vitro data showed that curcumin could effectively improve colistin-induced oxidative stress and apoptotic cell death in N2a neuronal cells by up-regulating the activities of the intracellular SOD, CAT and GSH levels, and promoting the activation of Nrf2/HO-1 pathway [26]. Curcumin treatment also downregulated the expression of NF-κB and NF-κB-regulated genes that are involved in the process of inflammatory response and apoptotic death in N2a cells [26]. In a rat model, oral administration of curcumin at the dose of 200 mg/kg/day for 6 days significantly improved colistin-induced inflammatory response, oxidative stress damage and apoptosis in the brain tissues (e.g., cerebral cortex and cerebellar cortex tissues) [27]. Our study revealed that oral administration of curcumin at the dose of 200 mg/kg/day for 7 days significantly ameliorated colistin-induced sciatic nerve damage by inhibiting oxidative stress, apoptosis and upregulating the nerve growth factor (NGF)/Akt and Nrf2/HO-1 pathways (Figure 5) [25]. Mechanically, NGF treatment promotes cell survival of nerve cells via the activation of Akt pathway [25]. The activation of Akt caused by NGF stimulation significantly attenuated colistin-induced apoptotic death and the inhibition of axon growth in N2a and PC12 neuronal cells, which involved the activation of Nrf2/ARE pathway [132,150,153]. Nerve growth factor plays a decisive role in promoting the growth and survival of peripheral sensory and sympathetic nerve cells [150]. Our recent study demonstrated that NGF administration could significantly inhibit colistin-induced peripheral neurotoxicity in mice [154]. Consistently, NGF treatment per se can inhibit colistin-induced oxidative stress and apoptosis in PC12 cells [150]. Thus, the protective effect of curcumin on colistin-induced peripheral neurotoxicity is partly attributed to the activation of NGF/Akt pathway.

## 6. Conclusions

During the past two decades, parenteral polymyxin B and colistin have been increasingly used for the treatment of problematic Gram-negative pathogens. Notwithstanding, neurotoxicity and nephrotoxicity remain the major dose-limiting factors hampering the clinical utility of these essential last-line antibiotics. Increasing reports of polymyxin resistance in the nosocomial setting and widespread environmental polymyxin resistance due to the plasmid mcr-1 has rattled the cage for the long-term therapeutic utility of polymyxins. Polymyxin combination treatments with FDA approved drugs and natural products is an emerging strategy for overcoming the aforementioned unwanted side-effects and bacterial resistance. Combining polymyxins with the existing drugs is prudent, in the view that FDA approved drugs have known pharmacokinetic and safety profiles; therefore, combination therapies could be fast-tracked into clinical practice. Curcumin is an FDA- approved natural product exhibiting many pharmacological antioxidant and healing activities. An increasing body of literature suggests that polymyxins–curcumin combination therapy has a synergistic inhibitory effect on the bacterial growth and lowers polymyxin-induced nephrotoxicity and neurotoxicity via inhibition of oxidative stress, mitochondrial dysfunction, inflammation and apoptosis. Future clinical trials of polymyxins–curcumin combination therapy are certainly warranted, albeit there is currently a dearth of data on optimal dosage regimens and pharmacokinetics for the combination, both of which require further studies.
